# [μ-*N*,*N*′-Bis(diphenyl­phosphinometh­yl)benzene-1,4-diamine-κ^2^ *P*:*P*′]bis­[(2,2′-bipyridine-κ^2^ *N*,*N*′)silver(I)] bis­(per­chlorate) acetone disolvate

**DOI:** 10.1107/S1600536809014871

**Published:** 2009-04-30

**Authors:** Liu-Cheng Gui, Qing-Ling Ni, Xuan-Feng Jiang, Jian-Qiang Zeng, Xiu-Jian Wang

**Affiliations:** a School of Chemistry and Chemical Engineering, Guangxi Normal University, Guilin 541004, People’s Republic of China

## Abstract

The title complex, [Ag_2_(C_10_H_8_N_2_)_2_(C_32_H_30_N_2_P_2_)](ClO_4_)_2_·2CH_3_COCH_3_, is a centrosymmetric dimer with pairs of Ag^I^ atoms bridged by *N*,*N*′-bis­(diphenyl­phosphinometh­yl)ben­zene-1,4-diamine ligands. In addition, each Ag^I^ atom is coordin­ated by one chelating 2,2′-bipyridine ligand, giving a distorted trigonal coordination environment.

## Related literature

Diphosphine ligands effectively stabilize low-valent *d* ^10^ metals complexes due to their electronic and steric characteristics, see: Meijboom *et al.* (2009 ▶); Ogasawara *et al.* (2000 ▶). Adducts of chelating polypyridyl ligands such as 2,2′-bipyridine and 1,10-phenanthroline (phen) always exhibit strong ligand–metal charge-transfer (LMCT) or metal–ligand charge-transfer (MLCT) absorption bands in the visible spectrum, see: Armaroli (2001 ▶). For a series of Ag^I^ and Cu^I^ complexes containing both diphosphine and chelating polypyridyl ligands which exhibit inter­esting photoluminescent properties at low temperature or even at room temperature, see: Wang *et al.* (2008 ▶). For the synthesis of *N*,*N*-bis­[(diphenyl­phosphino)meth­yl]-benzene-1,4-diamine, see: Durran *et al.* (2000 ▶); Hellmann *et al.* (1962 ▶). For related structures, see: Effendy *et al.* (2007 ▶); Zhang *et al.* (2003 ▶).
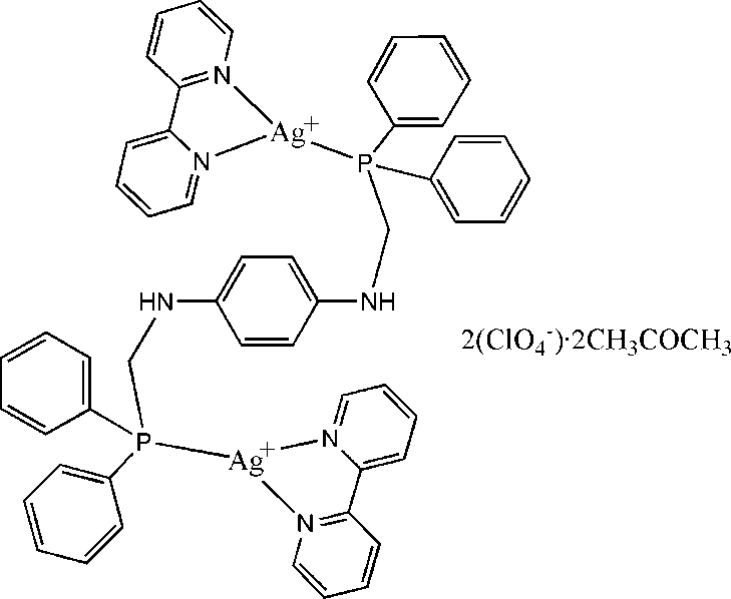


## Experimental

### 

#### Crystal data

[Ag_2_(C_10_H_8_N_2_)_2_(C_32_H_30_N_2_P_2_)](ClO_4_)_2_·2C_3_H_6_O
*M* *_r_* = 1347.68
Monoclinic, 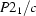

*a* = 10.6372 (13) Å
*b* = 15.8958 (19) Å
*c* = 16.6684 (19) Å
β = 91.432 (3)°
*V* = 2817.5 (6) Å^3^
*Z* = 2
Mo *K*α radiation
μ = 0.91 mm^−1^
*T* = 173 K
0.38 × 0.24 × 0.20 mm

#### Data collection

Bruker SMART CCD area-detector diffractometer
Absorption correction: multi-scan (*SADABS*; Sheldrick, 1998 ▶) *T*_min_ = 0.657, *T*_max_ = 0.833
14114 measured reflections
6120 independent reflections
3944 reflections with *I* > 2σ(*I*)
*R*_int_ = 0.044

#### Refinement

*R*[*F* ^2^ > 2σ(*F* ^2^)] = 0.046
*wR*(*F* ^2^) = 0.132
*S* = 1.05
6120 reflections
361 parameters
H-atom parameters constrained
Δρ_max_ = 1.02 e Å^−3^
Δρ_min_ = −0.68 e Å^−3^

### 

Data collection: *SMART* (Bruker, 1998 ▶); cell refinement: *SAINT* (Bruker, 1998 ▶); data reduction: *SAINT*; program(s) used to solve structure: *SHELXS97* (Sheldrick, 2008 ▶); program(s) used to refine structure: *SHELXL97* (Sheldrick, 2008 ▶); molecular graphics: *SHELXTL* (Sheldrick, 2008 ▶); software used to prepare material for publication: *SHELXTL*.

## Supplementary Material

Crystal structure: contains datablocks I, global. DOI: 10.1107/S1600536809014871/at2766sup1.cif

Structure factors: contains datablocks I. DOI: 10.1107/S1600536809014871/at2766Isup2.hkl

Additional supplementary materials:  crystallographic information; 3D view; checkCIF report

## Figures and Tables

**Fig. 1.**
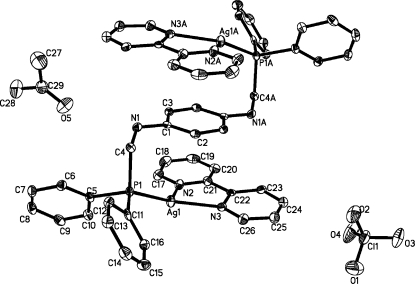
View of (I), showing 30% probability displacement ellipsoids and atom-numbering scheme for the contents of the asymmetric unit. [Symmetry code: (i) -x, y + 1/2, -z + 1/2]

## supplementary crystallographic information

### Comment

There has been great interest in d^10^ metals complexes containing diphosphine and polypyridyl ligands because of two main reasons: the first is diphosphine ligands effectively stabilize low-valent d^10^ metals complexes due to their electronic and steric characteristics (Meijboom *et al.*, 2009; Ogasawara *et al.*, 2000), and the other is the chelating polypyridyl ligands such as 2,2'-bipyridine and 1,10-phenanthroline (phen) are present of low-energy orbitals, the adducts always exhibit strong Ligand-metal charge-transfer (LMCT) or Metal-ligand charge-transfer (MLCT) absorption bands in the visible spectrum (Armaroli, 2001). In the past, we have obtained a series of Ag^I^ and Cu(I) complexes containing both diphosphine and chelating polypyridyl ligands, from which we found that these complexes all exhibit interesting photoluminescent properties at low temperature or even at room temperature (Wang *et al.*, 2008). Here we report the crystal and molecular structure of[Ag_2_(C_10_H_8_N_2_)_2_(C_32_H_30_N_2_P_2_)](ClO_4_)_2_.2CH_3_COCH_3_, (I). This work complements and extends our structural characterization of d^10^ metals complexes containing diphosphine and polypyridyl ligands.

### Experimental

The synthesis of (I) was carried out by the reaction of AgClO_4_.H_2_O (0.045 g, 0.2 mmol), 2,2'-bipyridine (0.032 g, 0.2 mmol) and *N*,*N*-bis[(diphenylphosphino)methyl]-benzene-1,4-diamine (0.0504 g, 0.1 mmol, synthesized according to literature (Durran, *et al.*, 2000; Hellmann *et al.*, 1962) in acetonitrile-acetone (7 ml with a ratio of 4: 3) solution, the resulting orange red solution was allowed to stir for 0.5 h at room temperature.Then by slow diffusion of diethyl ether into the solution, block red crystals were formed suitable for X-ray diffraction analysis.

### Refinement

All hydrogen atoms were generated geometrically with C—H = 0.95-0.99 Å and N—H = 0.88 Å, and refined with a riding model [U_iso_(H) = 1.2U_eq_(N,C)]. The highest residual peak is located 0.49Å from atom *Ag1* and the deepest hole is located 0.55Å from atom *Ag1*.

### Crystal data

**Table:**

[Ag_2_(C_10_H_8_N_2_)_2_(C_32_H_30_N_2_P_2_)](ClO_4_)_2_·2C_3_H_6_O	*F*(000) = 1372
*M**_r_* = 1347.68	*D*_x_ = 1.589 Mg m^−^^3^
Monoclinic, *P*2_1_/*c*	Mo *K*α radiation, λ = 0.71073 Å
Hall symbol: -P 2ybc	Cell parameters from 3856 reflections
*a* = 10.6372 (13) Å	θ = 2.3–25.9°
*b* = 15.8958 (19) Å	µ = 0.91 mm^−^^1^
*c* = 16.6684 (19) Å	*T* = 173 K
β = 91.432 (3)°	Block, red
*V* = 2817.5 (6) Å^3^	0.38 × 0.24 × 0.20 mm
*Z* = 2	

### Data collection

**Table:**

Bruker SMART CCD area-detector diffractometer	6120 independent reflections
Radiation source: fine-focus sealed tube	3944 reflections with *I* > 2σ(*I*)
graphite	*R*_int_ = 0.044
ω scans	θ_max_ = 27.1°, θ_min_ = 1.8°
Absorption correction: multi-scan (*SADABS*; Sheldrick, 1998)	*h* = −6→13
*T*_min_ = 0.657, *T*_max_ = 0.833	*k* = −19→20
14114 measured reflections	*l* = −21→18

### Refinement

**Table:**

Refinement on *F*^2^	Primary atom site location: structure-invariant direct methods
Least-squares matrix: full	Secondary atom site location: difference Fourier map
*R*[*F*^2^ > 2σ(*F*^2^)] = 0.046	Hydrogen site location: inferred from neighbouring sites
*wR*(*F*^2^) = 0.132	H-atom parameters constrained
*S* = 1.05	*w* = 1/[σ^2^(*F*_o_^2^) + (0.0655*P*)^2^] where *P* = (*F*_o_^2^ + 2*F*_c_^2^)/3
6120 reflections	(Δ/σ)_max_ < 0.001
361 parameters	Δρ_max_ = 1.02 e Å^−^^3^
0 restraints	Δρ_min_ = −0.68 e Å^−^^3^

### Special details

**Table:**

Geometry. All e.s.d.'s (except the e.s.d. in the dihedral angle between two l.s. planes) are estimated using the full covariance matrix. The cell e.s.d.'s are taken into account individually in the estimation of e.s.d.'s in distances, angles and torsion angles; correlations between e.s.d.'s in cell parameters are only used when they are defined by crystal symmetry. An approximate (isotropic) treatment of cell e.s.d.'s is used for estimating e.s.d.'s involving l.s. planes.
Refinement. Refinement of *F*^2^ against ALL reflections. The weighted *R*-factor *wR* and goodness of fit *S* are based on *F*^2^, conventional *R*-factors *R* are based on *F*, with *F* set to zero for negative *F*^2^. The threshold expression of *F*^2^ > σ(*F*^2^) is used only for calculating *R*-factors(gt) *etc*. and is not relevant to the choice of reflections for refinement. *R*-factors based on *F*^2^ are statistically about twice as large as those based on *F*, and *R*- factors based on ALL data will be even larger.

### Fractional atomic coordinates and isotropic or equivalent isotropic displacement parameters (Å^2^)

**Table:**

	*x*	*y*	*z*	*U*_iso_*/*U*_eq_	
Ag1	0.21755 (3)	0.40337 (2)	0.14606 (2)	0.03172 (13)	
Cl1	0.48508 (14)	0.89672 (9)	0.20183 (10)	0.0534 (4)	
P1	0.06386 (11)	0.32350 (7)	0.20844 (6)	0.0217 (2)	
O1	0.5433 (6)	0.8587 (4)	0.2698 (4)	0.124 (2)	
O2	0.5382 (5)	0.8626 (4)	0.1311 (4)	0.120 (2)	
O3	0.5114 (4)	0.9841 (3)	0.2031 (3)	0.0788 (14)	
O4	0.3559 (4)	0.8806 (3)	0.2030 (3)	0.0819 (15)	
O5	−0.2686 (4)	0.1981 (3)	0.0953 (3)	0.0705 (13)	
N1	−0.0972 (4)	0.3598 (2)	0.0811 (2)	0.0304 (9)	
H1A	−0.1320	0.3197	0.0517	0.037*	
N2	0.2913 (4)	0.4064 (2)	0.0167 (2)	0.0300 (8)	
N3	0.3101 (3)	0.5301 (2)	0.1275 (2)	0.0265 (8)	
C1	−0.0460 (4)	0.4297 (3)	0.0420 (2)	0.0231 (9)	
C2	−0.0201 (4)	0.5056 (3)	0.0812 (2)	0.0256 (10)	
H2A	−0.0332	0.5102	0.1372	0.031*	
C3	−0.0246 (4)	0.4255 (3)	−0.0395 (2)	0.0259 (10)	
H3A	−0.0407	0.3744	−0.0674	0.031*	
C4	−0.0954 (4)	0.3509 (3)	0.1662 (2)	0.0280 (10)	
H4A	−0.1557	0.3065	0.1810	0.034*	
H4B	−0.1235	0.4043	0.1905	0.034*	
C5	0.0783 (4)	0.2100 (3)	0.1927 (2)	0.0243 (9)	
C6	−0.0183 (5)	0.1549 (3)	0.2088 (2)	0.0297 (10)	
H6A	−0.0961	0.1757	0.2274	0.036*	
C7	−0.0017 (5)	0.0684 (3)	0.1979 (3)	0.0354 (12)	
H7A	−0.0685	0.0307	0.2086	0.042*	
C8	0.1110 (5)	0.0380 (3)	0.1719 (3)	0.0375 (12)	
H8A	0.1212	−0.0205	0.1628	0.045*	
C9	0.2093 (5)	0.0925 (3)	0.1589 (3)	0.0372 (12)	
H9A	0.2884	0.0710	0.1431	0.045*	
C10	0.1934 (5)	0.1786 (3)	0.1686 (3)	0.0320 (11)	
H10A	0.2610	0.2160	0.1589	0.038*	
C11	0.0445 (4)	0.3334 (2)	0.3160 (2)	0.0229 (9)	
C12	−0.0679 (4)	0.3110 (3)	0.3519 (3)	0.0284 (10)	
H12A	−0.1358	0.2891	0.3202	0.034*	
C13	−0.0799 (5)	0.3207 (3)	0.4338 (3)	0.0330 (11)	
H13A	−0.1567	0.3059	0.4581	0.040*	
C14	0.0169 (6)	0.3513 (3)	0.4800 (3)	0.0411 (13)	
H14A	0.0074	0.3579	0.5362	0.049*	
C15	0.1292 (5)	0.3730 (3)	0.4454 (3)	0.0414 (13)	
H15A	0.1972	0.3937	0.4777	0.050*	
C16	0.1419 (4)	0.3644 (3)	0.3634 (3)	0.0294 (10)	
H16A	0.2186	0.3800	0.3395	0.035*	
C17	0.2814 (5)	0.3429 (3)	−0.0352 (3)	0.0375 (12)	
H17A	0.2563	0.2892	−0.0164	0.045*	
C18	0.3065 (5)	0.3529 (4)	−0.1160 (3)	0.0444 (13)	
H18A	0.2973	0.3070	−0.1521	0.053*	
C19	0.3447 (5)	0.4298 (4)	−0.1426 (3)	0.0447 (14)	
H19A	0.3631	0.4378	−0.1975	0.054*	
C20	0.3565 (4)	0.4960 (3)	−0.0888 (3)	0.0355 (11)	
H20A	0.3844	0.5497	−0.1060	0.043*	
C21	0.3265 (4)	0.4821 (3)	−0.0092 (3)	0.0284 (10)	
C22	0.3353 (4)	0.5508 (3)	0.0512 (3)	0.0258 (10)	
C23	0.3680 (4)	0.6325 (3)	0.0318 (3)	0.0365 (12)	
H23A	0.3830	0.6474	−0.0224	0.044*	
C24	0.3788 (5)	0.6924 (3)	0.0920 (3)	0.0443 (13)	
H24A	0.4035	0.7483	0.0799	0.053*	
C25	0.3534 (5)	0.6699 (3)	0.1697 (3)	0.0402 (12)	
H25A	0.3601	0.7097	0.2120	0.048*	
C26	0.3187 (4)	0.5892 (3)	0.1843 (3)	0.0327 (11)	
H26A	0.2994	0.5740	0.2377	0.039*	
C27	−0.4843 (6)	0.1744 (5)	0.0964 (5)	0.087 (2)	
H27A	−0.4852	0.2307	0.1203	0.131*	
H27B	−0.5191	0.1338	0.1342	0.131*	
H27C	−0.5352	0.1743	0.0466	0.131*	
C28	−0.3312 (7)	0.0678 (4)	0.0406 (4)	0.075 (2)	
H28A	−0.2411	0.0606	0.0318	0.112*	
H28B	−0.3773	0.0651	−0.0110	0.112*	
H28C	−0.3607	0.0229	0.0758	0.112*	
C29	−0.3534 (6)	0.1507 (4)	0.0787 (3)	0.0477 (14)	

### Atomic displacement parameters (Å^2^)

**Table:**

	*U*^11^	*U*^22^	*U*^33^	*U*^12^	*U*^13^	*U*^23^
Ag1	0.0352 (2)	0.0282 (2)	0.0323 (2)	−0.00696 (17)	0.01181 (15)	0.00382 (16)
Cl1	0.0401 (8)	0.0428 (8)	0.0772 (10)	−0.0068 (6)	0.0019 (7)	−0.0166 (7)
P1	0.0262 (6)	0.0194 (5)	0.0198 (5)	−0.0024 (5)	0.0046 (5)	0.0026 (4)
O1	0.097 (5)	0.105 (4)	0.169 (6)	−0.012 (4)	−0.061 (5)	0.038 (4)
O2	0.073 (4)	0.116 (4)	0.173 (6)	−0.027 (3)	0.036 (4)	−0.097 (4)
O3	0.089 (4)	0.056 (3)	0.092 (3)	−0.036 (3)	0.017 (3)	−0.023 (2)
O4	0.035 (2)	0.070 (3)	0.142 (5)	−0.010 (2)	0.009 (3)	−0.023 (3)
O5	0.057 (3)	0.086 (3)	0.069 (3)	−0.019 (3)	0.002 (2)	−0.014 (2)
N1	0.038 (2)	0.029 (2)	0.0236 (19)	−0.0080 (18)	−0.0099 (17)	0.0009 (16)
N2	0.027 (2)	0.029 (2)	0.034 (2)	−0.0056 (17)	0.0079 (17)	−0.0015 (17)
N3	0.0234 (19)	0.029 (2)	0.0273 (19)	−0.0006 (16)	−0.0018 (16)	0.0013 (16)
C1	0.020 (2)	0.022 (2)	0.027 (2)	0.0009 (18)	−0.0072 (18)	0.0053 (17)
C2	0.026 (2)	0.031 (2)	0.019 (2)	0.002 (2)	−0.0001 (18)	0.0013 (18)
C3	0.032 (3)	0.023 (2)	0.022 (2)	0.0005 (19)	−0.007 (2)	−0.0028 (17)
C4	0.030 (3)	0.026 (2)	0.027 (2)	0.000 (2)	−0.002 (2)	0.0050 (19)
C5	0.033 (3)	0.022 (2)	0.019 (2)	0.0010 (19)	0.0032 (19)	0.0021 (17)
C6	0.034 (3)	0.030 (3)	0.025 (2)	−0.005 (2)	0.000 (2)	−0.0024 (19)
C7	0.054 (3)	0.026 (2)	0.026 (2)	−0.010 (2)	−0.005 (2)	0.0019 (19)
C8	0.060 (4)	0.021 (2)	0.031 (2)	0.001 (2)	0.004 (2)	−0.001 (2)
C9	0.042 (3)	0.034 (3)	0.036 (3)	0.015 (2)	0.004 (2)	0.001 (2)
C10	0.035 (3)	0.028 (3)	0.033 (2)	0.002 (2)	0.004 (2)	0.002 (2)
C11	0.033 (3)	0.012 (2)	0.025 (2)	0.0045 (18)	0.0110 (19)	0.0015 (17)
C12	0.029 (3)	0.028 (2)	0.028 (2)	−0.003 (2)	0.002 (2)	−0.0022 (19)
C13	0.041 (3)	0.035 (3)	0.023 (2)	−0.003 (2)	0.007 (2)	0.000 (2)
C14	0.065 (4)	0.035 (3)	0.023 (2)	0.008 (3)	0.001 (3)	−0.002 (2)
C15	0.050 (3)	0.046 (3)	0.027 (2)	0.001 (3)	−0.010 (2)	−0.002 (2)
C16	0.030 (3)	0.029 (2)	0.029 (2)	−0.004 (2)	0.000 (2)	0.003 (2)
C17	0.037 (3)	0.041 (3)	0.035 (3)	−0.006 (2)	0.002 (2)	−0.005 (2)
C18	0.042 (3)	0.056 (4)	0.035 (3)	−0.004 (3)	0.005 (2)	−0.013 (3)
C19	0.034 (3)	0.075 (4)	0.026 (3)	0.009 (3)	0.003 (2)	0.007 (3)
C20	0.028 (3)	0.045 (3)	0.034 (3)	0.000 (2)	0.004 (2)	0.008 (2)
C21	0.022 (2)	0.032 (3)	0.031 (2)	−0.002 (2)	0.0059 (19)	0.007 (2)
C22	0.017 (2)	0.030 (2)	0.030 (2)	−0.0009 (19)	−0.0068 (18)	0.0070 (19)
C23	0.031 (3)	0.039 (3)	0.039 (3)	−0.010 (2)	−0.013 (2)	0.012 (2)
C24	0.044 (3)	0.029 (3)	0.059 (3)	−0.006 (2)	−0.014 (3)	0.007 (2)
C25	0.034 (3)	0.032 (3)	0.054 (3)	0.000 (2)	−0.001 (3)	−0.002 (2)
C26	0.027 (2)	0.034 (3)	0.036 (3)	−0.002 (2)	0.006 (2)	−0.003 (2)
C27	0.057 (5)	0.088 (5)	0.119 (6)	0.012 (4)	0.033 (4)	0.029 (5)
C28	0.081 (5)	0.057 (4)	0.088 (5)	−0.012 (4)	0.024 (4)	−0.003 (4)
C29	0.047 (4)	0.051 (3)	0.045 (3)	−0.006 (3)	0.005 (3)	0.010 (3)

### Geometric parameters (Å, °)

**Table:**

Ag1—N3	2.267 (3)	C11—C16	1.378 (6)
Ag1—N2	2.314 (4)	C11—C12	1.397 (6)
Ag1—P1	2.3348 (11)	C12—C13	1.383 (6)
Cl1—O4	1.398 (4)	C12—H12A	0.9500
Cl1—O1	1.413 (6)	C13—C14	1.361 (7)
Cl1—O3	1.416 (4)	C13—H13A	0.9500
Cl1—O2	1.428 (5)	C14—C15	1.383 (7)
P1—C11	1.817 (4)	C14—H14A	0.9500
P1—C5	1.830 (4)	C15—C16	1.383 (6)
P1—C4	1.870 (4)	C15—H15A	0.9500
O5—C29	1.203 (6)	C16—H16A	0.9500
N1—C1	1.404 (5)	C17—C18	1.388 (7)
N1—C4	1.425 (5)	C17—H17A	0.9500
N1—H1A	0.8800	C18—C19	1.365 (8)
N2—C17	1.331 (6)	C18—H18A	0.9500
N2—C21	1.336 (5)	C19—C20	1.386 (7)
N3—C26	1.335 (5)	C19—H19A	0.9500
N3—C22	1.346 (5)	C20—C21	1.390 (6)
C1—C3	1.384 (6)	C20—H20A	0.9500
C1—C2	1.397 (6)	C21—C22	1.487 (6)
C2—C3^i^	1.387 (6)	C22—C23	1.386 (6)
C2—H2A	0.9500	C23—C24	1.387 (7)
C3—C2^i^	1.387 (6)	C23—H23A	0.9500
C3—H3A	0.9500	C24—C25	1.376 (7)
C4—H4A	0.9900	C24—H24A	0.9500
C4—H4B	0.9900	C25—C26	1.358 (6)
C5—C6	1.383 (6)	C25—H25A	0.9500
C5—C10	1.390 (6)	C26—H26A	0.9500
C6—C7	1.397 (6)	C27—C29	1.480 (8)
C6—H6A	0.9500	C27—H27A	0.9800
C7—C8	1.372 (7)	C27—H27B	0.9800
C7—H7A	0.9500	C27—H27C	0.9800
C8—C9	1.379 (7)	C28—C29	1.484 (8)
C8—H8A	0.9500	C28—H28A	0.9800
C9—C10	1.390 (6)	C28—H28B	0.9800
C9—H9A	0.9500	C28—H28C	0.9800
C10—H10A	0.9500		
			
N3—Ag1—N2	72.30 (12)	C13—C12—C11	119.7 (4)
N3—Ag1—P1	148.80 (9)	C13—C12—H12A	120.1
N2—Ag1—P1	133.15 (9)	C11—C12—H12A	120.1
O4—Cl1—O1	108.8 (4)	C14—C13—C12	120.7 (5)
O4—Cl1—O3	111.9 (3)	C14—C13—H13A	119.6
O1—Cl1—O3	109.0 (3)	C12—C13—H13A	119.6
O4—Cl1—O2	110.5 (3)	C13—C14—C15	120.2 (4)
O1—Cl1—O2	109.0 (4)	C13—C14—H14A	119.9
O3—Cl1—O2	107.6 (3)	C15—C14—H14A	119.9
C11—P1—C5	103.83 (17)	C14—C15—C16	119.6 (5)
C11—P1—C4	103.2 (2)	C14—C15—H15A	120.2
C5—P1—C4	104.8 (2)	C16—C15—H15A	120.2
C11—P1—Ag1	119.36 (15)	C11—C16—C15	120.7 (4)
C5—P1—Ag1	114.18 (14)	C11—C16—H16A	119.6
C4—P1—Ag1	110.07 (14)	C15—C16—H16A	119.6
C1—N1—C4	123.0 (4)	N2—C17—C18	122.0 (5)
C1—N1—H1A	118.5	N2—C17—H17A	119.0
C4—N1—H1A	118.5	C18—C17—H17A	119.0
C17—N2—C21	119.4 (4)	C19—C18—C17	118.9 (5)
C17—N2—Ag1	124.5 (3)	C19—C18—H18A	120.6
C21—N2—Ag1	115.1 (3)	C17—C18—H18A	120.6
C26—N3—C22	119.1 (4)	C18—C19—C20	119.5 (4)
C26—N3—Ag1	123.4 (3)	C18—C19—H19A	120.2
C22—N3—Ag1	116.3 (3)	C20—C19—H19A	120.2
C3—C1—C2	117.7 (4)	C19—C20—C21	118.5 (5)
C3—C1—N1	119.6 (4)	C19—C20—H20A	120.7
C2—C1—N1	122.7 (4)	C21—C20—H20A	120.7
C3^i^—C2—C1	120.9 (4)	N2—C21—C20	121.6 (4)
C3^i^—C2—H2A	119.5	N2—C21—C22	117.1 (4)
C1—C2—H2A	119.5	C20—C21—C22	121.2 (4)
C1—C3—C2^i^	121.4 (4)	N3—C22—C23	120.4 (4)
C1—C3—H3A	119.3	N3—C22—C21	116.7 (4)
C2^i^—C3—H3A	119.3	C23—C22—C21	122.8 (4)
N1—C4—P1	112.8 (3)	C22—C23—C24	119.4 (4)
N1—C4—H4A	109.0	C22—C23—H23A	120.3
P1—C4—H4A	109.0	C24—C23—H23A	120.3
N1—C4—H4B	109.0	C25—C24—C23	119.2 (5)
P1—C4—H4B	109.0	C25—C24—H24A	120.4
H4A—C4—H4B	107.8	C23—C24—H24A	120.4
C6—C5—C10	119.5 (4)	C26—C25—C24	118.4 (5)
C6—C5—P1	122.1 (3)	C26—C25—H25A	120.8
C10—C5—P1	118.3 (3)	C24—C25—H25A	120.8
C5—C6—C7	120.1 (4)	N3—C26—C25	123.4 (4)
C5—C6—H6A	120.0	N3—C26—H26A	118.3
C7—C6—H6A	120.0	C25—C26—H26A	118.3
C8—C7—C6	120.1 (5)	C29—C27—H27A	109.5
C8—C7—H7A	120.0	C29—C27—H27B	109.5
C6—C7—H7A	120.0	H27A—C27—H27B	109.5
C7—C8—C9	120.1 (4)	C29—C27—H27C	109.5
C7—C8—H8A	120.0	H27A—C27—H27C	109.5
C9—C8—H8A	120.0	H27B—C27—H27C	109.5
C8—C9—C10	120.3 (5)	C29—C28—H28A	109.5
C8—C9—H9A	119.9	C29—C28—H28B	109.5
C10—C9—H9A	119.9	H28A—C28—H28B	109.5
C9—C10—C5	119.9 (4)	C29—C28—H28C	109.5
C9—C10—H10A	120.0	H28A—C28—H28C	109.5
C5—C10—H10A	120.0	H28B—C28—H28C	109.5
C16—C11—C12	119.0 (4)	O5—C29—C27	119.9 (6)
C16—C11—P1	119.6 (3)	O5—C29—C28	122.0 (6)
C12—C11—P1	121.3 (3)	C27—C29—C28	118.2 (6)

Symmetry codes: (i) −*x*, −*y*+1, −*z*.
